# Supplementary material for: Screening and Research on Skin Barrier Damage Protective Efficacy of Different Mannosylerythritol Lipids
Source: Molecules. 2022 Jul 21;27(14):4648. doi: 10.3390/molecules27144648 (PMC9320248; doi:10.3390/molecules27144648)

Supplementary Materials

Screening and Research on Skin Barrier Damage Protective Efficacy of Different Mannosylerythritol Lipids.

Chenxu Jing [1, 2, †], Jiling Guo [2, †], Zhenzhuo Li [1, †], Xiaohao Xu [1, 2], Jing Wang [1], Lu Zhai [1], Jianzeng Liu [2], Guang Sun [1], Fei Wang [1], Yangfen Xu [3], Zhaolian Li [3], Daqing Zhao [2] , Rui Jiang [1, *] and Liwei Sun [1, *]

[1] *Research Center of Traditional Chinese Medicine, The Affiliated Hospital to Changchun University of Chinese Medicine, Changchun, Jilin Province, China;*

[2] *Jilin Ginseng Academy, Changchun University of Chinese Medicine, Changchun, Jilin Province, China;*

[3] *Modern Hanfang Technology Company Limited, Guangzhou, Guangdong Province, China*

[*] Correspondence: jiangrui800710@163.com (R.J.); sunnylilwei@163.com (L.S.)

[†] These authors contributed equally to this work.

Table of Contents

Figure S1: HRESIMS of **MEL-A**.

Figure S2: ^{1}H NMR spectrum of **MEL-A** in $CDCl_3$ (500 MHz).

Figure S3: ^{13}C NMR and DEPT spectrum of **MEL-A** in $CDCl_3$ (126 MHz).

Figure S4: HRESIMS of **MEL-B**.

Figure S5: ^{1}H NMR spectrum of **MEL-B** in $CDCl_3$ (500 MHz).

Figure S6: ^{13}C NMR and DEPT spectrum of **MEL-B** in $CDCl_3$ (126 MHz).

Figure S7: HRESIMS of **MEL-C**.

Figure S8: ^{1}H NMR spectrum of **MEL-C** in $CDCl_3$ (600 MHz).

Figure S9: ^{13}C NMR and DEPT spectrum of **MEL-C** in $CDCl_3$ (151 MHz).

Figure S1: HRESIMS of **MEL-A**

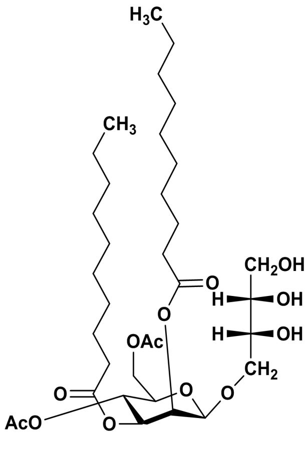

MEL-A

$C_{34}H_{60}O_{13}$

W.M. 676.4034

Qualitative Analysis Report

**Data Filename**	HEML01.d	**Sample Name**	HEML01
**Sample Type**	Sample	**Position**	P1-A2
**Instrument Name**	Instrument 1	**User Name**	
**Acq Method**	s.m	**Acquired Time**	7/13/2018 10:07:16 AM
**IRM Calibration Status**	Success	**DA Method**	Default.m
**Comment**			

Sample Group **Info.**

Acquisition SW Version 6200 series TOF/6500 series Q-TOF B.05.01 (B5125.2)

User Spectra

Fragmentor Voltage	Collision Energy	Ionization Mode
135	0	ESI

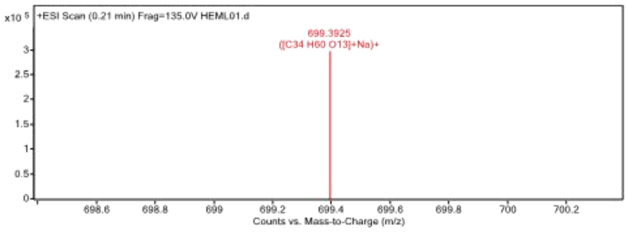

Peak List

m/z	z	Abund	Formula	Ion
629.3505	1	87145.12		
657.3816	1	151115.89		
666.4055	1	91860.55		
671.3612	1	433487.25		
672.3646	1	152958.31		
687.335	1	142183.25		
697.3768	1	85202.36		
699.3925	1	297716.16	C34 H60 O13	(M+Na)+
700.3961	1	107502.94	C34 H60 O13	(M+Na)+
715.3665		104703.45		

Formula Calculator Element Limits

Element	Min	Max
C	3	60
H	0	120
O	0	30

Formula Calculator Results

Formula	CalculatedMass	CalculatedMz	Mz	Diff. (mDa)	Diff. (ppm)	DBE
C34 H60 O13	676.4034	699.3926	699.3925	0.10	0.14	5.0000

--- End Of Report ---

Figure S2: ^{1}H NMR spectrum of **MEL-A** in $CDCl_3$ (500 MHz).

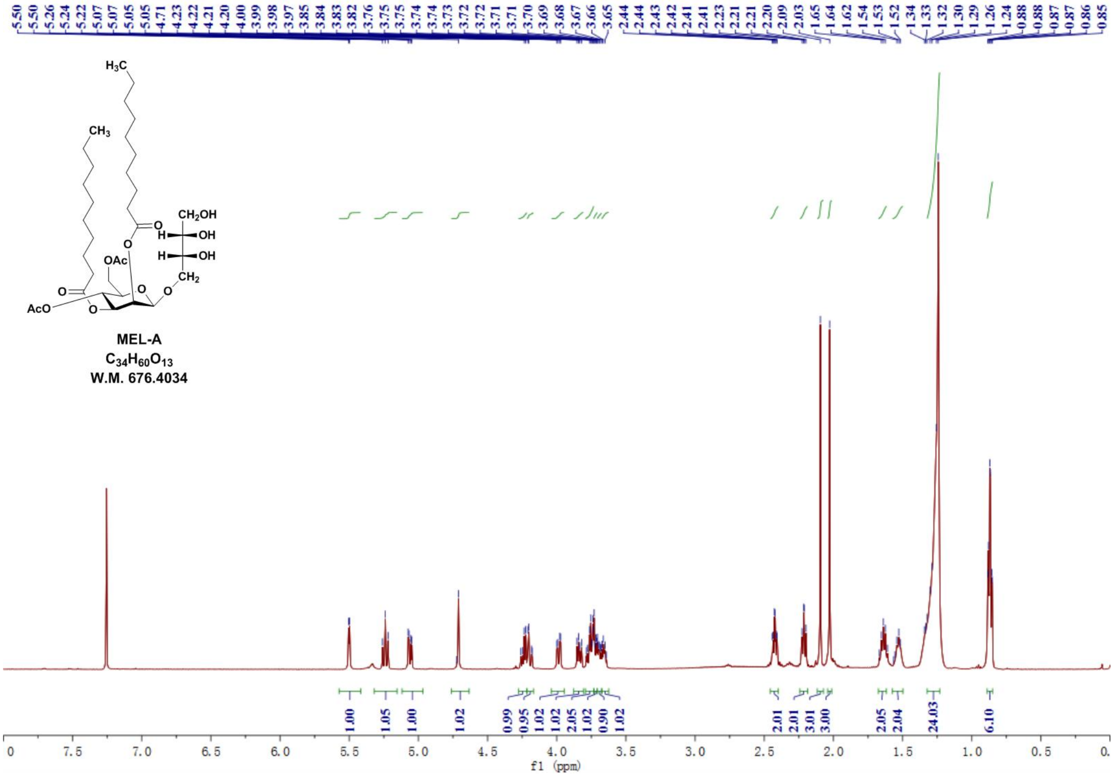

Figure S3: ^{13}C and DEPT NMR spectrum of **MEL-A** in $CDCl_3$ (126 MHz).

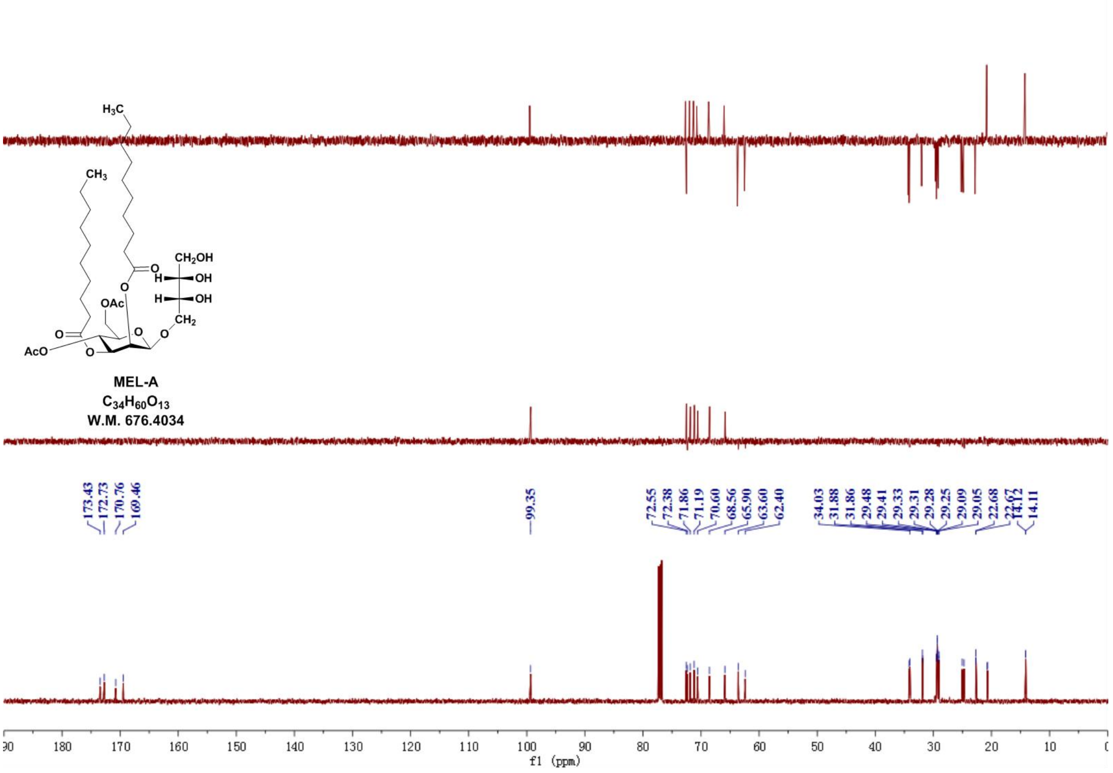

Figure S4: HRESIMS of **MEL-B**

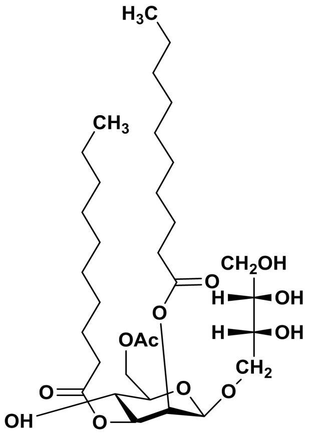

MEL-B
$C_{32}H_{58}O_{12}$
W.M. 634.3928

Qualitative Analysis Report

**Data Filename**	HEML03.d	**Sample Name**	HEML03
**Sample Type**	Sample	**Position**	P1-A3
**Instrument Name**	Instrument 1	**User Name**	
**Acq Method**	s.m	**Acquired Time**	7/13/2018 10:08:27 AM
**IRM Calibration Status**	Success	**DA Method**	Default.m
**Comment**			

Sample Group **Info.**
Acquisition SW 6200 series TOF/6500 series
Version Q-TOF B.05.01 (B5125.2)

User Spectra

Fragmentor Voltage	Collision Energy	Ionization Mode
135	0	ESI

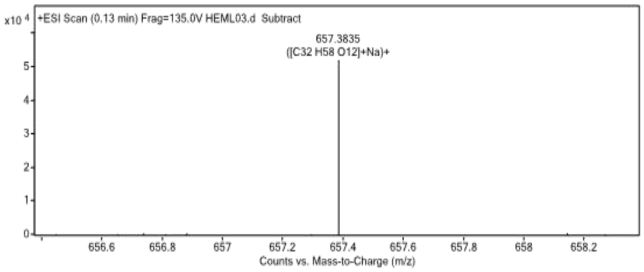

Peak List

m/z	z	Abund	Formula	Ion
102.129	1	81070.5		
274.2751	1	27670.45		
318.3018	1	27710.97		
629.3521	1	28813.09		
657.3835	1	52066.29	C32 H58 O12	(M+Na)+
671.3623	1	50138.6		
673.3585	1	26043.09		
687.337	1	24854.12		
699.3941	1	34777.34		
715.3686	1	21239.64		

Formula Calculator Element Limits

Element	Min	Max
C	3	60
H	0	120
O	0	30

Formula Calculator Results

Formula	CalculatedMass	CalculatedMz	Mz	Diff. (mDa)	Diff. (ppm)	DBE
C32 H58 O12	634.3928	657.3820	657.3835	-1.50	-2.28	4.0000

--- End Of Report ---

Figure S5: ^{1}H NMR spectrum of **MEL-B** in $CDCl_3$ (500 MHz).

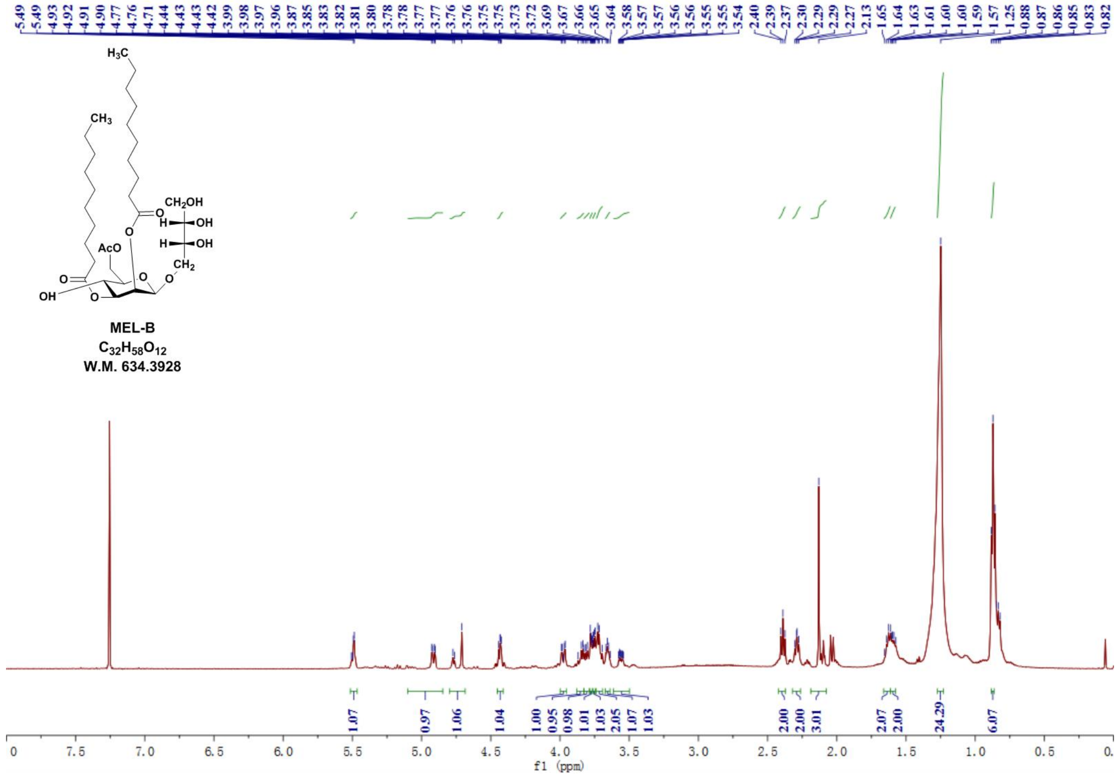

Figure S6: ^{13}C and DEPT NMR spectrum of **MEL-B** in $CDCl_3$ (126 MHz).

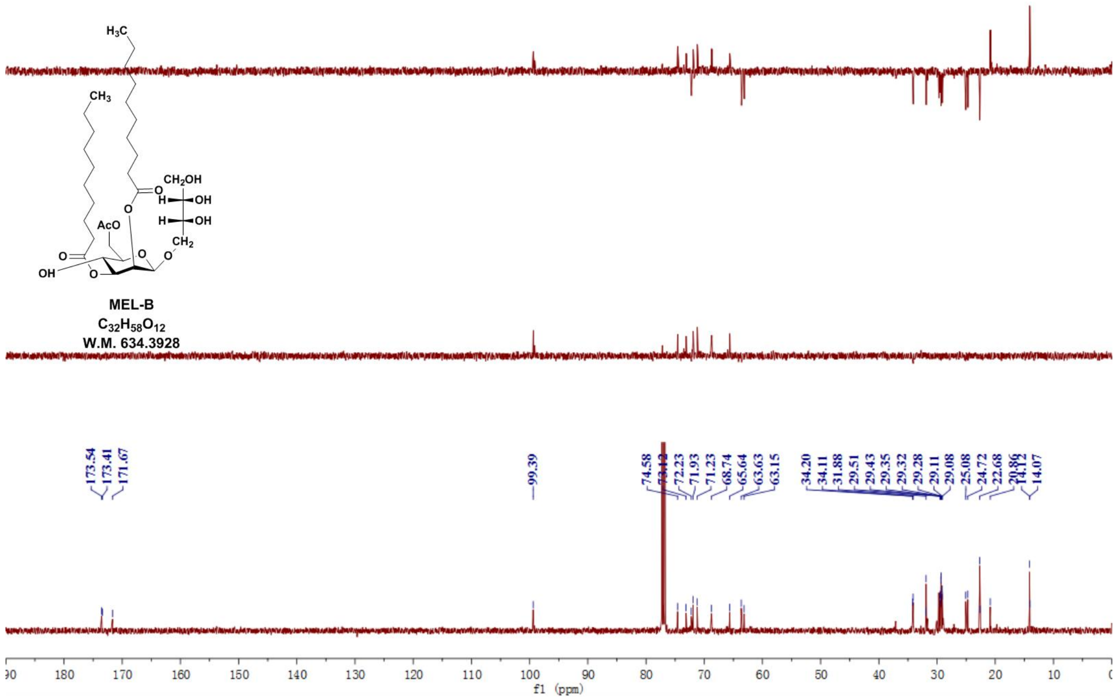

Figure S7: HRESIMS of **MEL-C**

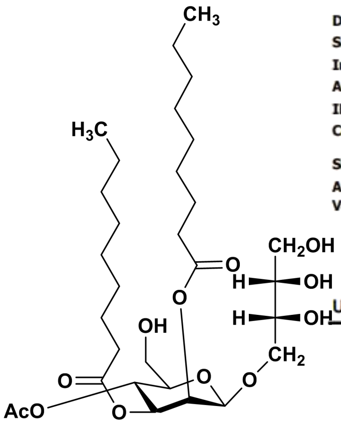

MEL-C
$C_{30}H_{54}O_{12}$
W.M. 606.3615

Qualitative Analysis Report

**Data Filename**	HMEL10-1.d	**Sample Name**	HMEL10-1
**Sample Type**	Sample	**Position**	P1-A1
**Instrument Name**	Instrument 1	**User Name**	
**Acq Method**	s.m	**Acquired Time**	9/9/2020 2:36:49 PM
**IRM Calibration Status**	Success	**DA Method**	Default.m
**Comment**			

**Sample Group**		**Info.**
**Acquisition SW**	6200 series TOF/6500 series	
**Version**	Q-TOF B.05.01 (B5125.2)	

User Spectra

Fragmentor Voltage	Collision Energy	Ionization Mode
250	0	ESI

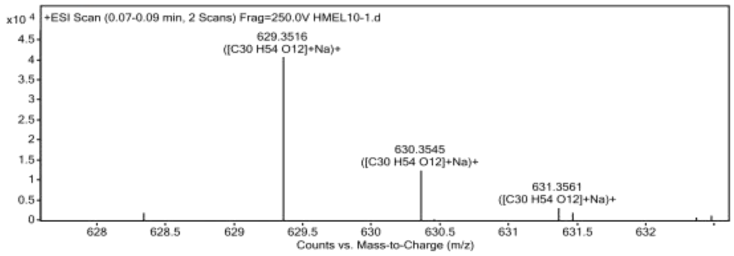

Peak List

m/z	z	Abund
545.2579	1	73056.17
657.383	1	107804.38
671.3625	1	241115.44
672.3656	1	81602.03
697.378	1	88028.43
699.3933	1	537962.63
700.3971	1	196029.84
701.3994	1	44330.29
715.3669	1	45312.07
809.5022	1	44510.05

Formula Calculator Element Limits

Element	Min	Max
C	3	60
H	0	120
O	0	30

Formula Calculator Results

Formula	CalculatedMass	CalculatedMz	Mz	Diff. (mDa)	Diff. (ppm)	DBE
C30 H54 O12	606.3615	629.3507	629.3516	-0.90	-1.43	4.0000

--- End Of Report ---

Figure S8: ^{1}H NMR spectrum of **MEL-C** in $CDCl_3$ (600 MHz).

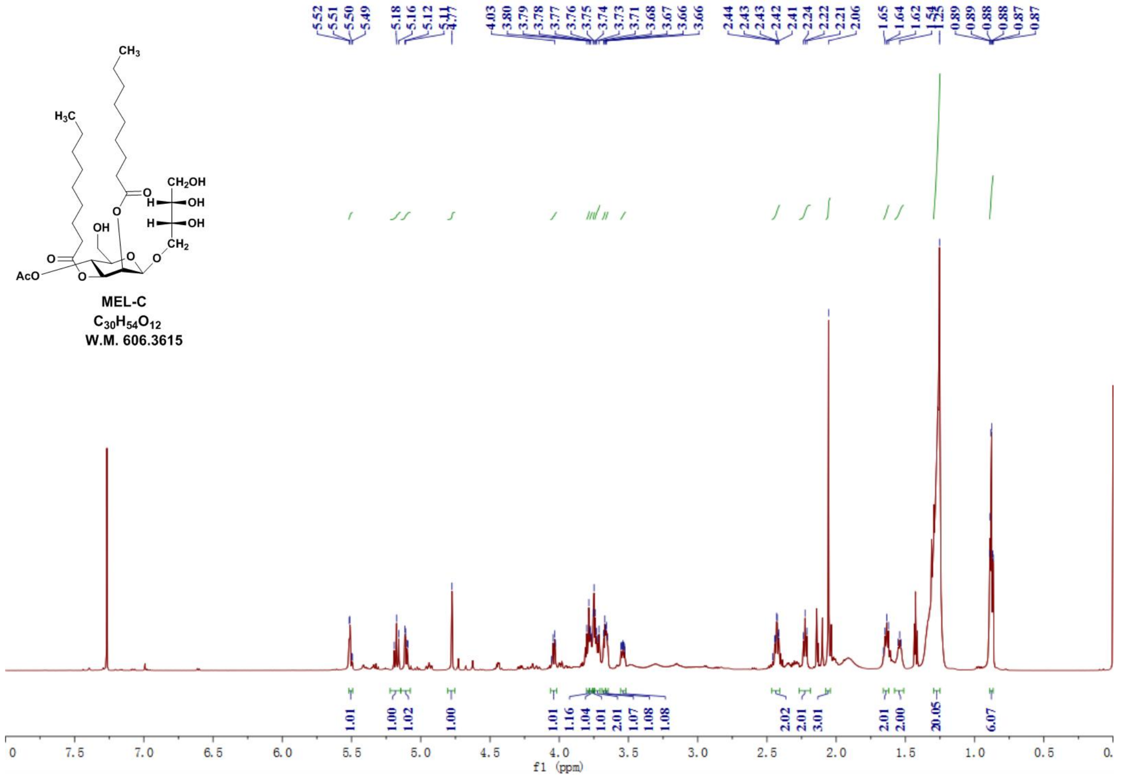

Figure S9: ^{13}C and DEPT NMR spectrum of **MEL-C** in $CDCl_3$ (151 MHz).

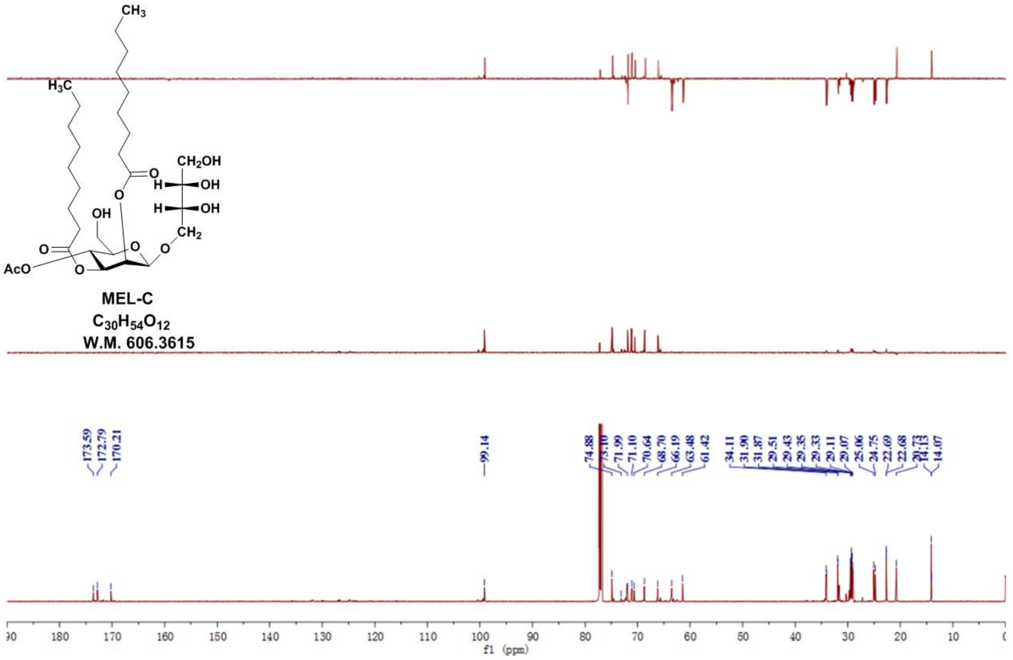

Supplement: Supplementary file 1 [file molecules-27-04648-s001.zip › molecules-1769463-supplementary.pdf]
